# Functional characterization of NADPH-cytochrome P450 reductase and cinnamic acid 4-hydroxylase encoding genes from *Scoparia dulcis* L.

**DOI:** 10.1186/s40529-020-00284-4

**Published:** 2020-03-02

**Authors:** Yoshimi Yamamura, Ayaka Mabuchi

**Affiliations:** Faculty of Pharmaceutical Sciences, University of Toyama, 2630 Sugitani, Toyama, Toyama 930-0194 Japan

**Keywords:** Cinnamic acid 4-hydroxylase, NADPH-cytochrome P450 reductase, P450, *Scoparia dulcis* L.

## Abstract

**Background:**

Most plant cytochrome P450 (P450) proteins need to be supplied with electrons from a redox partner, e.g. an NADPH-cytochrome P450 reductase (CPR), for the activation of oxygen molecules via heme. CPR is a flavoprotein with an N-terminal transmembrane domain, which transfers electrons from NADPH to the P450 via coenzymes flavin adenine dinucleotide and flavin mononucleotide.

**Results:**

In this study, a novel CPR (*SdCPR*) was isolated from a tropical medicinal plant *Scoparia dulcis* L. The deduced amino acid of SdCPR showed high homology of > 76% with CPR from higher plants and belonged to the class II CPRs of dicots. Recombinant SdCPR protein reduced cytochrome c, ferricyanide (K_3_Fe(CN)_6_), and dichlorophenolindophenol in an NADPH-dependent manner. To elucidate the P450 monooxygenase activity of SdCPR, we isolated a cinnamic acid 4-hydroxylase (*SdC4H*, CYP73A111) gene from *S. dulcis*. Biochemical characterization of SdCPR/SdC4H demonstrated that SdCPR supports the oxidation step of SdC4H. Real-time qPCR results showed that expression levels of *SdCPR* and *SdC4H* were inducible by mechanical wounding treatment and phytohormone elicitation (methyl jasmonate, salicylic acid), which were consistent with the results of promotor analyses.

**Conclusions:**

Our results showed that the SdCPR and SdC4H are related to defense reactions, including the biosynthesis of secondary metabolites.

## Background

Cytochrome P450 (P450) proteins are heme-containing monooxygenases that are distributed in a wide range of organisms ranging from bacteria to mammals. Higher plants have a large number of P450 molecular species compared with other organisms, which is considered to reflect the diversity of metabolism in plants (Rana et al. 2013). In fact, plant P450s are involved in various secondary metabolic biosynthesis reactions, including reactions involving fatty acids, phenylpropanoids, alkaloids, and the biosynthesis and metabolism of plant hormones. The phenylpropanoid pathway involves a common oxidation reaction, p-hydroxylation of cinnamic acid catalyzed by P450 from the CYP73 family (Additional file 1: Fig. S1). Eukaryotic P450s contain membrane anchored peptides, which the N-terminus directs targeting to the endoplasmic reticulum (ER) (Rana et al. 2013). The catalytic activity of P450 depends on electron supply from a redox partner NADPH-cytochrome P450 reductase (CPR) (Rana et al. 2013). CPRs transport electrons from NADPH first to flavin adenine dinucleotide (FAD), then to flavin mononucleotide (FMN), and finally to P450 heme. Genes encoding CPRs have been isolated from animals, insects and yeast, and so far they are all present as a single copy and interact with various P450s (Porter et al. 1990). In contrast, it has been reported that higher plants have one to three copies of CPR (Rana et al. 2013). In addition, Ro et al. (Ro et al. 2002) classified CPRs into two classes, class I and class II, based on N-terminal sequences. CPRs classified in class I have a short N-terminal sequence and are constitutively expressed in plants, whereas class II CPRs are expressed in response to stress or elicitors/injury.

*Scoparia dulcis* L. (Plantaginaceae) is a perennial herb widely distributed in tropical areas. Several unique diterpenes (ex, scopadulcic acid B [SDB] and scopadulciol etc.) have been isolated from *S. dulcis* and have been shown to have various biological activities (Hayashi 2000). Hayashi et al. demonstrated that the biosynthesis of SDB is markedly activated in *Scoparia* leaf tissues by treatment with methyl jasmonate (MJ) (Hayashi et al. 1999; Nkembo et al. 2006). We recently discovered novel candidate genes (encoding diterpene synthase and P450) potentially involved in SDB biosynthesis by transcriptome analysis (Yamamura et al. 2017).

P450s are membrane proteins that requires a redox partner for expression of their enzyme activity; therefore, preparation of recombinant P450 proteins has been mainly performed in eukaryotic expression systems, such as yeast (Yamamura et al. 2001; Hayashi et al. 2007) and insect cells (Ohnishi et al. 2012). Recently, several strategies have been developed for the expression of eukaryotic P450s in *Escherichia coli* (prokaryotic expression system) to characterize their activities (Hausjell et al. 2018). This approach may be applied to elucidate the various biosynthetic mechanisms of secondary metabolism in higher plants.

In this study, we isolated a CPR essential for the activity of P450 from *S. dulcis*. The isolated *Scoparia* CPR (SdCPR) was further characterized for a monooxygenase activity with *Scoparia* cinnamic acid 4-hydroxylase P450 (SdC4H; CYP73A111) in vitro. The expression patterns of *SdCPR* and the *SdC4H* were also examined in *Scoparia* leaves after treatment with elicitor and wounding.

## Materials and methods

### Plant material and treatments

*Scoparia dulcis* L. were grown in sterile conditions on half-strength Murashige and Skoog plates at 25 °C in continuous light. Eight-week-old plants were used for all experiments. All plant leaves were evenly sprayed (three times) with 0.1 mM aqueous solutions of MJ (Sigma-Aldrich, MO, USA) and salicylic acid (SA; Nacalai Tesque, Kyoto, Japan), which were pre-dissolved in 99% ethanol. After incubation for 0–6 h, the second leaves from the top (fully expanded leaf) were used for qPCR. For mechanical wounding treatment, second leaves were cut into 2-mm fragments and incubated for 1–8 h in a petri dish (floated on 10 mL distilled water containing 0.005% (w/v) chloramphenicol). Samples were collected and frozen immediately in liquid nitrogen and stored at − 80 °C.

### Cloning of *SdCPR* and *SdC4H* genes

Total RNA was extracted from *S. dulcis* leaves using TRIzol (Thermo Fisher Scientific, Waltham, MA, USA) in accordance with the manufacturer’s instructions. First strand cDNA was synthesized using a PrimeScript II 1st strand cDNA Synthesis Kit (Takara Bio Inc., Kusatsu, Shiga, Japan). cDNAs of *SdCPR* and *SdC4H* were isolated using degenerate primers (Additional file 1: Table S1). 5′- and 3′-end amplifications were carried out using a 5′ and 3′ rapid amplification of cDNA ends Kit, 2nd Generation (Roche Diagnostics GmbH, Mannheim, Germany) in accordance with the manufacturer’s instructions. The PCR products were subcloned into a pGEM-T easy vector (Promega, Madison, WI, USA). All DNA sequences of PCR-amplified open reading frames (ORFs) were confirmed using an ABI 3130 Genetic Analyzer (Applied Biosystems, Waltham, MA, USA).

### Heterologous expression of SdCPR and SdC4H in *E*. *coli*

The ORFs of *SdCPR* and *SdC4H* were amplified using Pwo DNA polymerase (Roche). The PCR products were inserted into the expression vector pET28b (Merck Millipore, Burlington, MA, USA) using an In-fusion HD Cloning Kit (Takara Bio Inc.). *E. coli* BL21 (DE3) cells harboring the expression vector were grown overnight in LB medium with 50 μg mL^−1^ kanamycin and 1% glucose at 37 °C in a shaking incubator, then diluted 1:25 into fresh LB medium supplemented with 50 μg mL^−1^ kanamycin. Cells were grown at 37 °C at 200 rpm until absorbance at 600 nm reached 0.4 − 0.6, and then 1 mM isopropyl β-D-1-thiogalactopyranoside (IPTG) was added. The culture was shaking at 200 rpm at 25 °C overnight for protein expression. The bacterial cells were collected by centrifugation at 3000 rpm for 5 min at 4 °C and washed twice with 4 °C wash buffer (10 mM Tris–HCl [pH 7.5], 150 mM NaCl). Then, the washed cell pellet was suspended in the BugBuster Protein Extraction Reagent (Novagen-Merck Millipore) and His-tag recombinant proteins were purified from the supernatant using MagneHis Ni-Particles (Promega) with elution buffer containing 1 M imidazole.

### Recombinant enzyme assays

The activities of SdCPR was assayed as described by Yang et al. (Yang et al. 2010). The assay was performed in a Hitachi U-2000A UV spectrophotometer, and reduction of cytochrome c was monitored by the increase in absorbance at 550 nm, at 25 °C, in 50 mM Tris buffer, pH 7.4, containing 100 μM cytochrome c and 100 μM NADPH. The reaction was started by the addition of NADPH. A molar absorption coefficient of 21 mM^−1^ cm^−1^ for cytochrome c was used for quantification. Reduction of dichlorophenol indophenol (DCPIP) was monitored at 600 nm (20.6 mM^−1^ cm^−1^), ferricyanide (K_3_Fe(CN)_6_) at 424 nm (1.02 mM^−1^ cm^−1^). To determine kinetic the parameters for cytochrome c, 100 μM NADPH was added to the reaction mixtures containing varying concentrations of cytochrome c. The kinetic parameters for NADPH were measured using 100 μM cytochrome c with varying NADPH concentrations. The substrate concentration for half maximal activity (K_m_) and maximum rate of reaction (V_max_) values were obtained using Hanes–Woolf plot analysis. In vitro C4H enzyme assays were initiated by adding 2 mM NADPH to the reaction mixture (1 mL total volume) containing 50 mM phosphate buffer (pH 7.4), 1 mM *trans*-cinnamic acid (Wako, Osaka, Japan), 50 μg recombinant SdCPR soluble fraction, and 100 μg recombinant SdC4H soluble fraction. After incubation at 30 °C for 30 min, the reaction was stopped by adding 67 μL 6 M HCl, and the reaction mixture was extracted three times with 500 μL of EtOAc, followed by evaporation of the organic phase in vacuo. The residues were dissolved in 600 μL of MeOH and analyzed using n high-performance liquid chromatography system (Hitachi High-Technologies Co., Tokyo, Japan), based on the method described by Ro et al. (Ro et al. 2001).

### Promoter cloning and analysis

The *SdCPR* and *SdC4H* promoter sequences (5′ untranslated leader regions) were obtained using a Universal GenomeWalker 2.0 Kit (Takara Bio Inc.). The PCR products were cloned into a pGEM-T easy vector and then sequenced. PlantCare (http://bioinformatics.psb.ugent.be/webtools/plantcare/html/) (Lescot et al. 2002) and PLACE (https://www.dna.affrc.go.jp/PLACE/?action=newplace) (Higo et al. 1999) were used for identification of *cis*-elements.

### Real-time qPCR

Real-time qPCR was performed using Brilliant III Ultra-Fast SYBR Green QPCR Master Mix (Agilent Technologies, Santa Clara, CA, USA) on an Mx3005p real-time QPCR system (Agilent Technologies). The *S. dulcis GAPDH* gene (JF718777) was used for normalization. The primer sequences used in the qPCR study are listed in Additional file 1: Table S1. Calibration curves were produced for each of the primer pairs and quantification was performed using the MxPro software (Agilent Technologies). Each sample was tested three times and each mRNA expression value was expressed as mean ± standard deviation (SD).

### Homology modelling and prediction of 3-D structure of SdCPR

The 3-D structure of SdCPR was constructed using the PHYRE2 server (http://www.sbg.bio.ic.ac.uk/~phyre2/html/page.cgi?id=index) (Kelley et al. 2015) using the crystal structure of *Rattus norvegicus* CPR (PDB ID: 1J9Z) as a template. Protein model refinement was performed using KoBaMIN server2012 (http://chopra-modules.science.purdue.edu/modules/kobamin/html/). Structurally, evolutionary, and functionally important regions were identified in deduced protein sequence by ConSurf (https://consurf.tau.ac.il/). Topology of the modelled SdCPR protein was analyzed using PDBSum (http://www.ebi.ac.uk/thornton-srv/databases/cgi-bin/pdbsum/GetPage.pl?pdbcode=index.html).

## Results

### Isolation of a full-length cDNA of CPR from *S. dulcis*

**Fig. 1:**
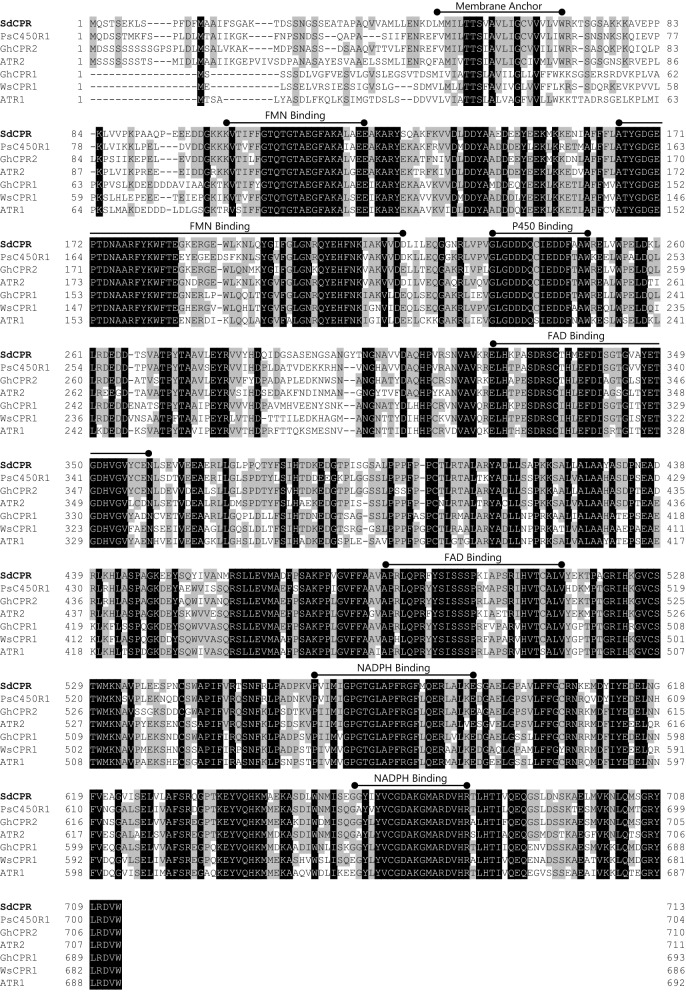
Alignment of the deduced amino acid sequences of SdCPR and plant P450 reductases. The deduced amino acid sequences of SdCPR was aligned with those of CPRs from *Pisum sativum* (PsC405R1), *Gossypium hirsutum* (GhCPR2), *Arabidopsis thaliana* (ATR2), *G*. *hirsutum* (GhCPR1), *Withania somnifera* (WsCPR1), and *A*. *thaliana* (ATR1) using the ClustalW program. Descriptions of CPRs used in the alignment are listed in Additional file 1: Table S2. The conserved regions and binding sites are marked

Based on the conserved region of a previously isolated plant CPR, degenerate primers were designed for the P450- and NADPH-binding region, which are highly conserved motifs in the amino acid sequence of higher plant derived CPRs. PCR was performed using cDNA prepared from *Scoparia* leaves as a template. A full-length CPR cDNA was obtained and named *SdCPR* (Accession number: KF306080). The nucleotide sequence of *SdCPR* contained an ORF of 2142 bp, and a predicted 713-amino acids protein sequence (estimated molecular weight: 78.5 kDa, PI: 5.09). The SdCPR ORF had conserved binding domains for FMN, FAD, NADPH, and P450, and the membrane anchor was present at the N-terminus (Fig. 1). The SdCPR protein sequence shared 77% sequence identity with pea (*Pisum sativum*, PsC450R1) and 67% with ashwagandha (*Withania somnifera*, WsCPR1) as well as 64% and 74% with Arabidopsis (*Arabidopsis thaliana*, ATR1 and ATR2), and 68% and 77% with cotton (*Gossypium hirsutum*, GhCPR1 and GhCPR2).

**Fig. 2:**
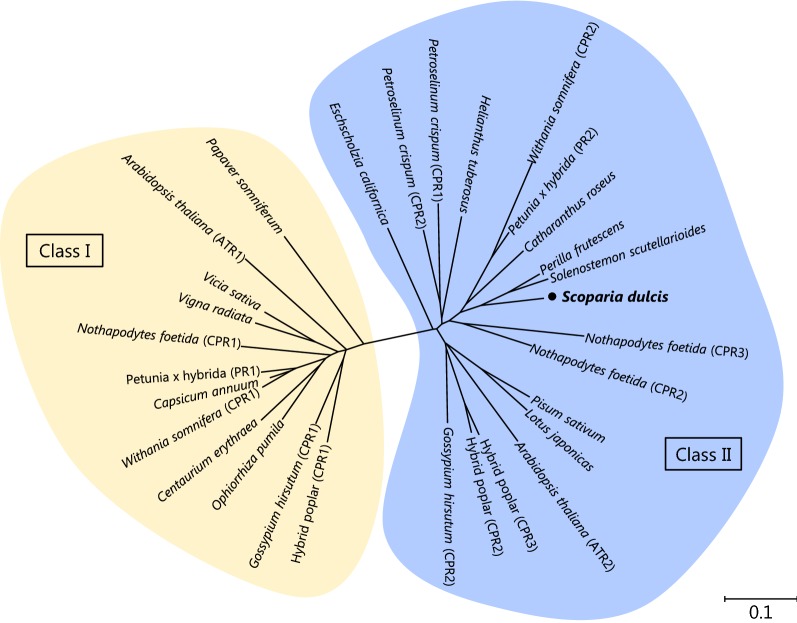
Phylogenetic trees of SdCPR. The maximum likelihood trees illustrate the phylogenetic relatedness of SdCPR with other CPRs. Descriptions of CPRs used in the phylogeny are listed in Additional file 1: Table S2. Phylogenetic analyses were performed using the neighbor-jointing method using Genetyx ver.14 software. The scale bar indicates the number of amino acid substitutions per site

CPRs can be classified into class I and class II based on the length of the N-terminal hydrophobic region (Ro et al. 2002). The N-terminal sequences of GhCPR1, WsCPR1, and ATR1 (belonging to class I CPRs) were revealed to be shorter sequence. In contrast, SdCPR contained a Ser/Thr rich extended N-terminal region, like other class II CPRs (PsC450R1, GhCPR2, and ATR2) (Fig. 1). Phylogenetic analysis also showed that SdCPR belong to class II group (Fig. 2). In addition, DNA blotting analysis showed that a single copy of *SdCPR* was present in the *S. dulcis* genome (Additional file 1: Fig. S2), and the result was identical to our transcriptome analysis (Yamamura et al. 2017).

### Heterologous overexpression and catalytic parameters of recombinant SdCPR

To examine the catalytic activity of SdCPR, the *SdCPR* gene was subcloned into pET-28b and used to transform *E. coli* BL21 (DE3) cells. The recombinant SdCPR protein was confirmed by immunoblotting analysis (Additional file 1: Fig. S3). The recombinant SdCPR was assayed for cytochrome c reduction activities in the presence of NADPH or NADH. The recombinant SdCPR showed cytochrome c reduction activity in an NADPH-dependent manner (Table 1); however, this activity was not detected in the absence of NADPH (data not shown). Cytochrome c activity was not observed in the presence of NADH (Table 1). Subsequently, the reduction in activity of the recombinant SdCPR against various electron acceptors was examined. Cytochrome c, DCPIP, and K_3_Fe(CN)_6_ were all active as electron acceptors (Table 2). The kinetic parameters K_m_ and V_max_ of SdCPR for NADPH and cytochrome c are shown in Table 3. The K_m_ and V_max_ of SdCPR were 4.6 ± 0.9 μM and 2.3 ± 0.1 μmol min^−1^ mg protein^−1^ for NADPH, 2.7 ± 0.6 μM and 2.5 ± 0.1 μmol min^−1^ mg protein^−1^ for cytochrome c (Table 3).

**Table 1:** Cytochrome c reductase reaction of SdCPR

	Specific activity (μmol/min/mg protein)	
	NADPH	NADH
SdCPR	2.14 ± 0.08	ND

Specific activity of SdCPR in reducing cytochrome c (100 μM), in the presence of 100 μM of NADPH or NADH (n = 3). Value are presented as mean ± SE

*ND* not detected

**Table 2:** Reduction of cytochrome c, K_3_Fe(CN)_6_, and DCPIP

	Specific activity (μmol/min/mg protein)		
	Cytochrome c	DCPIP	K_3_Fe(CN)_6_
SdCPR	2.14 ± 0.08	1.90 ± 0.05	8.58 ± 0.76

Reduction of cytochrome c and DCPIP at 100 μM, and K_3_Fe(CN)_6_ at 100 μM, by recombinant SdCPR (n = 3). Value are presented as mean ± SE

**Table 3:** Steady-state kinetic constant of cytochrome c and NADPH

	NADPH	Cytochrome c
V_max_ (μmol/min/mg^−1^)	2.3 ± 0.1	2.5 ± 0.1
K_m_ (μM)	4.6 ± 0.9	2.7 ± 0.6
k_cat_ (min^−1^)	177.6 ± 7.8	198.1 ± 6.6
k_cat_ K_m_^−1^	39.9 ± 6.4	77.2 ± 16.4

Steady-state kinetic constants of recombinant SdCPR at 28 ℃, pH 7.5. Determination of kinetic parameters for cytochrome c was performed in reaction mixture containing 100 μM NADPH and various amounts of cytochrome c, and kinetic parameters for NADPH was determined by using 100 μM cytochrome c as substrate and various amounts of NADPH (n = 3). Value are presented as mean ± SE

### SdCPR supported P450 monooxygenase activity

**Fig. 3:**
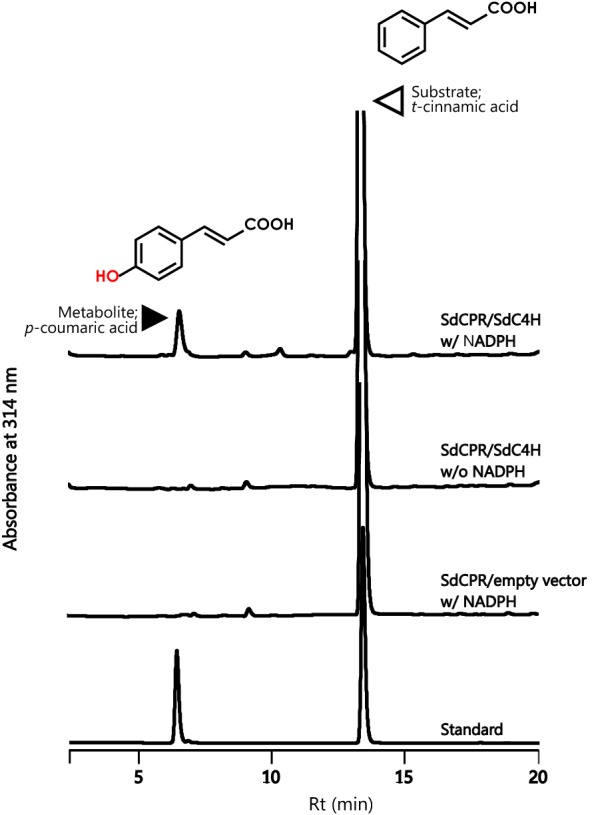
HPLC analysis of 4-hydroxylase assays. *E. coli* cell lysates containing recombinant SdCPR and SdC4H (SdCPR/SdC4H) with NADPH (w/NADPH) or without NADPH (w/o NADPH). Empty vector (SdCPR/vector) with NADPH. Authentic standards of *p*-coumaric acid (Rt = 6.5 min) and *t*-cinnamic acid (Rt = 13.0 min) (Standard)

In order to show P450 monooxygenase activity as support for SdCPR, we cloned a novel cinnamic acid 4-hydroxylase (Additional file 1: Fig. S1) from *S. dulcis* (designated as *SdC4H*, Accession number: KF306081, Additional file 1: Fig S4 and S5). SdC4H was named CP73A111 by the Committee on Cytochrome P450 Nomenclature (Nelson 2009). The full-length ORF of *SdC4H* was inserted into pET28b, and the construct was used to transform *E. coli* BL21(DE3) cells and expression induced by IPTG (Additional file 1: Fig. S3). The crude fraction was incubated with recombinant SdCPR and substrate *trans*-cinnamic acid. In the presence of NADPH, the C4H activity (trans-cinnamic acid was 4-hydroxylated) of recombinant SdC4H was detected by HPLC (Fig. 3). In contrast, no product formation was observed in assays without NADPH and vector only (Fig. 3). These results suggested that SdCPR is efficient in supporting SdC4H (CYP73A111) activity.

### Promoter analysis

Generally, it is known that class I CPRs are expressed constitutively, whereas the expression of class II CPRs is inducible by stress or elicitor exposure (Zhao et al. 2018). Because SdCPR belonged to class II according to phylogenetic analysis, this suggested that *SdCPR* expression may be induced by stress and elicitors. Increased *C4H* gene expression due to wounding, elicitors, or pathogen infection has also been reported in many plants (Chapple 1998). For these reasons, we searched for phytohormone- and stress-related *cis*-acting elements upstream of *SdCPR* and *SdC4H*. Several putative phytohormone- and stress-inducible *cis*-elements were identified in the promoters of *SdCPR* and *SdC4H*, which included a wounding-inducible WUN-motif and W-box, MJ/SA-responsive CGTCA and TGACG motif, and abscisic acid-responsive ABRE motif, DPBF binding site motif and MYB2A (Table 4).

**Table 4:** Putative *cis*-acting elements in the *SdCPR* and the *SdC4H* promotors related in phytohormone and stress responses

Motif	*SdCPR*	*SdC4H*	Function	Source
AAAC-motif	1	−	Light responsive	PlantCARE
A-box	−	1	Elicitor or light responsive	PlantCARE
ABRE motif	1	1	ABA responsive	PlantCARE
ARE	3	1	Anaerobic inducible	PlantCARE
ATCT-motif	2	1	Light responsive	PlantCARE
AuxRR-core	−	1	Auxin responsive	PlantCARE
Box 4	1	3	Light responsive	PlantCARE
CGTCA-motif	2	1	MeJA-responsive	PlantCARE
G-box	1	1	Light responsive	PlantCARE
GC-motif	1	1	Anoxic specific inducible	PlantCARE
GT1-motif	2	−	Light responsive	PlantCARE
I-box	1	−	Light responsive	PlantCARE
MBS	−	1	Drought inducible	PlantCARE
Sp1	2	−	Light responsive	PlantCARE
TCCC-motif	1	−	Light responsive	PlantCARE
TGACG-motif	2	1	MeJA and SA responsive	PlantCARE
WUN-motif	2	−	Wounding responsive	PlantCARE
DPBF binding site motif	2	2	ABA inducible	PLACE
E-box	8	6	Drought inducible	PLACE
ERE	1	−	Ethylene responsive	PLACE
GCC box	2	−	Elicitor responsive	PLACE
GT-1 motif	6	1	Pathogen and salt stress responsive	PLACE
MYB2AT	2	−	ABA inducible	PLACE
W-box	1	2	Wounding and fungal elicitor responsive	PLACE

“−” indicates absence of the motif; The number indicates number of times of occurrence of the motif

### Spatial distribution of *SdCPR* and *SdC4H* gene transcripts in *S. dulcis*

**Fig. 4:**
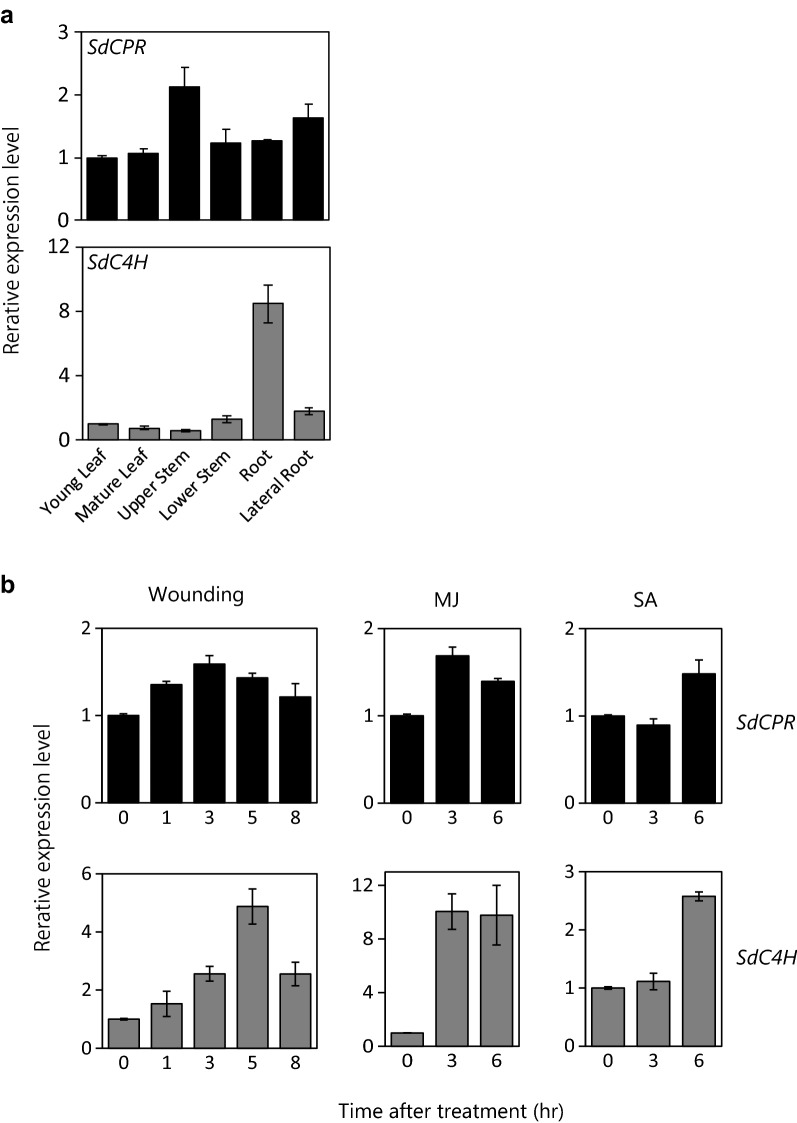
Real-time qPCR analysis of *SdCPR* and *SdC4H* genes. **a** Tissue-specific accumulation levels of the *SdCPR* and *SdC4H* genes in *S. dulcis*. Each organ was harvested from 8-week-old *Scoparia* plants for the isolation of total RNA. The transcript levels of each gene in young leaves were set to 1.0. **b** Effect of various treatments on expression levels of the *SdCPR* and *SdC4H* genes. The third leaves of *Scoparia* plant were treated with wounding, 0.1 mM methyl jasmonate (MJ) and 0.1 mM salicylic acid (SA). The transcript levels of each genes in the leaf at 0 h were set to 1.0. Each data was normalized to an internal control (*GAPDH*), and the ΔΔC_T_ method was used to obtain relative values. Error bars represent the ± SD of the mean (n = 3)

The organ specificities of the *SdCPR* and *SdC4H* genes in *S. dulcis* were analyzed by qPCR. *SdCPR* transcripts were detected at almost the same levels in all organs (Fig. 4a). In contrast, *SdC4H* transcripts were observed to increase especially in the roots. The expression levels in the roots were approximately eightfold higher than those found in other organs (Fig. 4a).

### Effect of wounding and MJ and SA on *SdCPR* and *SdC4H* gene expression levels

From promotor analysis, we speculated that the expression levels of *SdCPR* and *SdC4H* are more likely to be inducible by wounding and elicitors. Therefore, we further investigated the genes expression patterns of *SdCPR* and *SdC4H* in *S. dulcis* leaves after mechanical wounding and elicitor treatments. *SdCPR* mRNA levels were increased 1.5-fold within 3 − 6 h after wounding and MJ/SA treatment (Fig. 4b). *SdCPR* expression was significantly enhanced by wounding, increasing 1.5-fold within 3 h after wounding treatment. *SdC4H* transcript levels were significantly enhanced within the first 1 h after wounding, 3 h after MJ treatment, and 6 h after SA elicitation (Fig. 4b). There was a time correlation between changes in the expression of both genes. Our results indicated that *SdCPR* and *SdC4H* expression levels were induced in response to wounding and elicitor (MJ and SA), which were consistent with the identified *cis*-elements.

### Prediction of 3-D structure

**Fig. 5:**
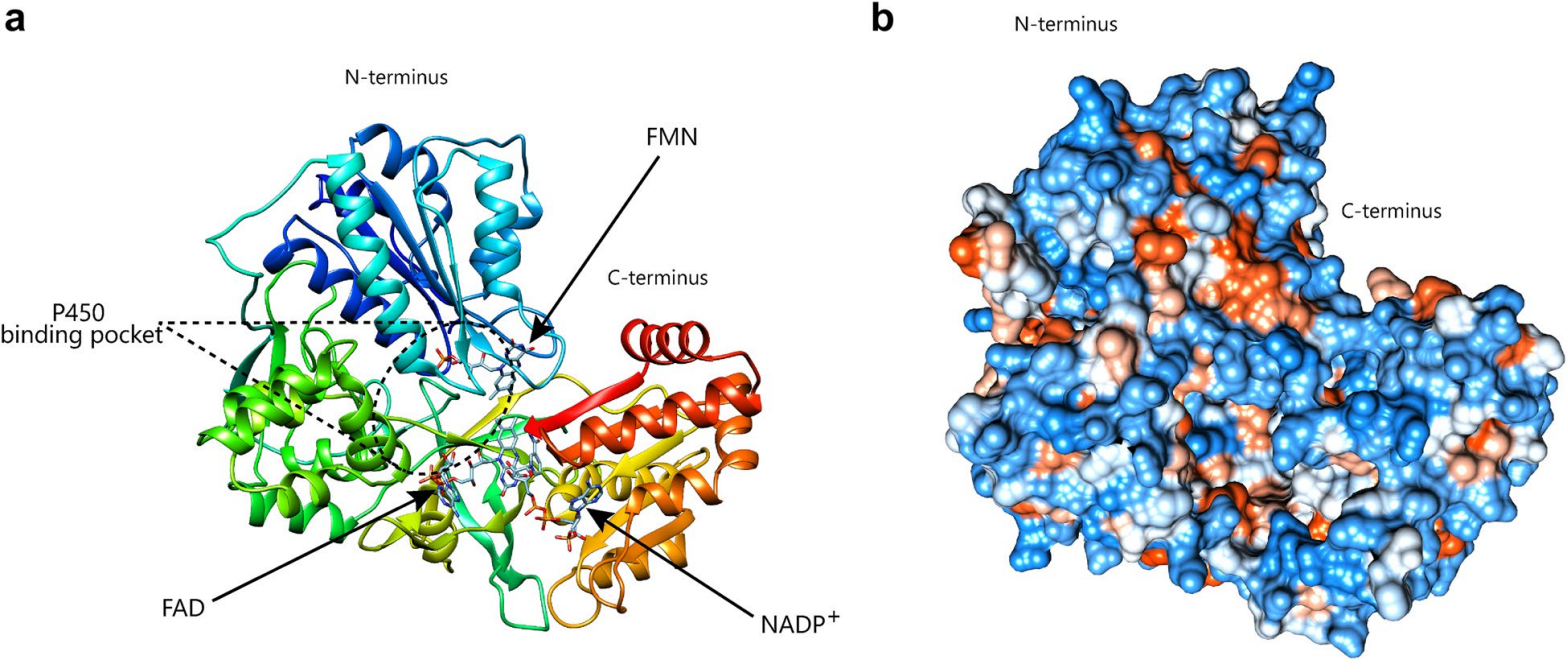
3-D model and conserved residue prediction for SdCPR. **a** Consensus matching of SdCPR as ribbon-structures. Ribbon display of the 3-D structures of SdCPR as predicted by PHYRE2 web server using crystal structure *Rattus norvegicus* (PDB ID: 1J9Z) as template. **b** Conserved residue analysis of SdCPR

Based on the structure of *Rattus norvegicus* CPR (PDB ID: 1J9Z), a predicted 3-D structure of SdCPR was constructed using a bioinformatics tool (Fig. 5). The P450 binding pocket was also presented in the predicted 3-D structure of SdCPR (Fig. 5a). Subsequently, docking experiments with FMN, FAD, and NADP^+^ were conducted to investigate the positional relationship at the active center. FMN, FAD, and NADP^+^ molecules were all located in the active pocket, and it was speculated that they play an important role in the reaction of P450 (Fig. 5a). The amino acid residues with high scores (in red) were functional and structural residues of SdCPR (Fig. 5b).

## Discussion

CPRs are membrane bound proteins localized in the ER, and they function to transfer electrons from NADPH through FAD and FMN to the heme iron center of the various P450 enzymes. It was reported that only one CPR gene is present in yeast, insects, and animals (Porter et al. 1990). The CPR only serves as a redox partner to support various P450 functions in yeast, insects, and animals. On the other hand, filamentous fungi possess one to multiple CPRs, and P450-CPR fusion enzymes have been discovered in some species (Lah et al. 2008). Higher plants also contain one to three paralogs of CPRs with different amino acid lengths and regulatory mechanisms (Rana et al. 2013). For instance, two CPRs from Arabidopsis, cotton, ashwagandha, and centaury (*Centaurium erythraea*) belong to class I and class II groups, respectively (Mizutani and Ohta 1998; Schwarz et al. 2009; Yang et al. 2010; Rana et al. 2013). Class I CPRs are involved in growth, development, and metabolism; therefore, these CPRs are constitutively expressed in plants (Yang et al. 2010). On the other hand, class II CPRs have been implicated in plant defense systems against environmental stresses. In some plant species, only one CPR gene has been reported, such as in coleus (*Solenostemon scutellarioides*), perilla (*Perilla frutescens*), *Catharanthus roseus*, *Lotus japonicus*, pea, and *Croton stellatopilosus*, which are categorized as class II CPRs (Meijer et al. 1993; Brosché et al. 1999; Eberle et al. 2009; Sintupachee et al. 2015; Fujiwara and Ito 2017). It is assumed that *S. dulcis* has only one CPR gene from DNA blotting (Additional file 1: Fig. S2) and transcriptome analyses (Yamamura et al. 2017). The mRNA of *SdCPR* was detected in all tissues of *S. dulcis* plants (Fig. 4a), indicating that the only one SdCPR is widely expressed in *S. dulcis* to support oxidation reactions involving over 200 P450s in metabolism.

The reductase activity of cytochrome c by recombinant SdCPR was clearly dependent on NADPH but not on NADH. Similarly, cytochrome c was reduced by CPRs from mung bean (*Vigna radiata*), poplar (*Populus trichocarpa* x *Populus deltoids*), parsley (*Petroselinum crispum*), and cotton in an NADPH-dependent manner (Shet et al. 1993; Koopmann and Hahlbrock 1997; Ro et al. 2002; Yang et al. 2010). On the other hand, house fly (*Musca domestica*) CPR catalysis of cytochrome c reduction involves NADH as an electron donor (Murataliev et al. 1999). Of note, Döhr et al. reported that the substitution of human CPR Trp-676 with alanine resulted in an enzyme that had about 1000-fold higher specificity for NADH than the wild-type enzyme (Döhr et al. 2001). This data establishes an important role for Trp-676 in NADH binding and recognition, which may provide a functional NADH-dependent P450 monooxygenase system.

Plant P450s play an important role in the biosynthesis of secondary metabolites and are often induced by various stresses. In previous reports, it was demonstrated that the biosynthesis of SDB, a tetracyclic diterpene in *S. dulcis*, is markedly activated by the MJ and yeast extract treatments (Nkembo et al. 2005; Yamamura et al. 2014). It is clear that a large number of P450s are responsible for not only SDB biosynthesis but also in the other biosynthetic reactions of secondary metabolites in *S. dulcis* (Yamamura et al. 2017). Among P450s, C4H is a key enzyme in phenylpropanoid biosynthetic pathways such as PAL and 4CL (Additional file 1: Fig. S1) and is known to be inducible by wounding and elicitors (Dixon and Paiva 1995; Bell-Lelong et al. 1997; Mizutani et al. 1997; Akashi et al. 1998). Similarly, Arabidopsis *ATR2* expression was induced by wounding and light stress (Mizutani and Ohta 1998), and cotton *GhCPR2* expression was inducible by wounding and fungal elicitor treatment (Yang et al. 2010). Two CPRs (ATR2 and GhCPR2) belonging to class II were induced by stress or elicitors and are likely to be involved in secondary metabolism (Zhao et al. 2018). Based on these reports, we attempted to prove that expression of *SdCPR* and *SdC4H* is inducible by stress. The results showed that a variety of elements related to different stress responses such as defense, light, elicitor treatment, and wounding were observed in both the *SdCPR* and *SdC4H* promotor regions (Table 4). In support of this result, both gene transcripts were strongly enhanced in response to different types of stresses such as wounding and MJ and SA treatment (Fig. 4b). Therefore, it is suggested that the SdCPR and SdC4H play an important role in stress-induced defense responses in *S. dulcis*.

## Conclusions

We isolated and characterized a novel NADPH-P450 reductase from *S. dulcis*, which is member of the class II CPRs. SdCPR activities in reducing cytochrome c, DCPIP, and K_3_Fe(CN)_6_, and in supporting P450 monooxygenase (SdC4H) were determined using recombinant proteins produced in *E. coli*. Expression analysis indicated that both *SdCPR* and *SdC4H* transcripts were induced by elicitor treatment and wounding, which was fully consistent with the identified promoter *cis*-elements. SdCPR may be helpful to clarify the SDB biosynthetic mechanisms involving multiple P450s in *S. dulcis*. Our study established a platform to characterize the P450s involved in plant metabolism.

## Supplementary information

**Additional file 1: Table S1.** Primers used in this study. **Table S2.** GenBank ID of CPRs in Figs. 1 and  2. **Figure S1.** The reaction catalyzed by C4H in the phenylpropanoid pathway. **Figure S2:** DNA blotting analysis of *SdCPR* by digesting with *Bgl*II, *Hin*dIII, and *Xba*I. **Figure S3.** Immunoblotting analysis of heterologously expressed His-SdCPR and His-SdC4H in *E. coli*. **Figure S4.** Amino acid alignment of the plant CYP73A family. **Figure S5.** Phylogenetic tree of P450 proteins involved in the phenylpropanoid pathway.

## Data Availability

Not applicable.

## Abbreviations

C4H: Cinnamic acid 4-hydroxylase
CPR: NADPH-cytochrome P450 reductase
DCPIP: Dichlorophenolindophenol
ER: Endoplasmic reticulum
GAPDH: Glyceraldehyde-3-phosphate dehydrogenase
IPTG: Isopropyl β-D-1-thiogalactopyranoside
K_3_Fe(CN)_6_: Ferricyanide
MJ: Methyl jasmonate
P450: Cytochrome P450
qPCR: Quantitative PCR
SA: Salicylic acid
SDB: Scopadulcic acid B
