# Supplementary material for: Functional characterization of NADPH-cytochrome P450 reductase and cinnamic acid 4-hydroxylase encoding genes from Scoparia dulcis L
Source: Bot Stud. 2020 Mar 2;61:6. doi: 10.1186/s40529-020-00284-4 (PMC7052086; doi:10.1186/s40529-020-00284-4)
Supplement: Supplementary file 1 — Additional file 1: Table S1. Primers used in this study. Table S2. GenBank ID of CPRs in Figs. 1 and 2. Figure S1. The reaction catalyzed by C4H in the phenylpropanoid pathway. Figure S2: DNA blotting analysis of SdCPR by digesting with BglII, HindIII, and XbaI. Figure S3. Immunoblotting analysis of heterologously expressed His-SdCPR and His-SdC4H in E. coli. Figure S4. Amino acid alignment of the plant CYP73A family. Figure S5. Phylogenetic tree of P450 proteins involved in the phenylpropanoid pathway. [file 40529_2020_284_MOESM1_ESM.docx]

Additional file 1

**Functional characterization of NADPH-cytochrome P450 reductase and cinnamic acid 4-hydroxylase encoding genes from *Scoparia dulcis* L.**

Yoshimi Yamamura* and Ayaka Mabuchi
Faculty of Pharmaceutical Sciences, University of Toyama,

2630 Sugitani, Toyama, Toyama 930-0194, Japan.

Content

**Table S1:** Primers used in this study.

**Table S2:** GenBank ID of CPRs in Fig. 1 and Fig. 2.

**Figure S1:** The reaction catalyzed by C4H in the phenylpropanoid pathway.

**Figure S2:** DNA blotting analysis of *SdCPR* by digesting with *Bgl*II, *Hin*dIII, and *Xba*I.

**Figure S3:** Immunoblotting analysis of heterologously expressed His-SdCPR and His-SdC4H in *E. coli*.

**Figure S4:** Amino acid alignment of the plant CYP73A family.

**Figure S5:** Phylogenetic tree of P450 proteins involved in the phenylpropanoid pathway.

**Table S1. Primers used in this study.**

| Primer | Sequences (5’ - 3’) |
| --- | --- |
| ***Degenerate*** | |
| CPR-FW2 | GAYCARWSHATWGARGATGA |
| CPR-RV1 | CTTTCYTGIARRAAWCCYCT |
| CYP73A-FW2 | TTYGGHAACTGGCTHCARGTYGGVGAYGA |
| CYP73A-RV1 | AGCTTNGCRTCRTGDAGGTTCATRTG |
| ***5’ and 3’ RACE*** | |
| b-19-FW2 | CTATTCCATCTCATCCTCACCA |
| EcoRI-d(T)_15_ | GCGCGCGAATTCTTTTTTTTTTTTTTT |
| SdCPR-RV2 | CATGTGAGTGCAAGAACGGTCAGA |
| A4 | ATAGAACCTAGCAGCATTGTCG |
| 3’-FW1 | TTCGTCTCCGGATGGCCATT |
| 5’-RV2 | AGGAGACGACGACGAGGTT |
| ***Full length*** | |
| SdCPR-ORF-Fw | ATGCAATCCACATCGGAGAA |
| SdCPR-Rv0 | CCAGACATCACGCAAGTACC |
| SdCYP73A-ORF-Fw | ATGGATCTTCTCCTACTCGAGA |
| SdCYP73A-ORF-Rv | TCAAAATGATCTAGGCTTCAA |
| ***DNA blot*** | |
| Fw-S1 | CAATGCTGTGGTTGATGC |
| SdCPR-RV0 | CCAGACATCACGCAAGTACC |
| ***Heterologous expression*** | |
| SdCPR-Sal-Fw2 | TTCGAGCTCCGTCGACATGCAATCCACATCGGAGAAGCTG |
| SdCPR-Xho-Rv2 | GGTGGTGGTGCTCGAGTCACCAGACATCACGCAAGTAC |
| SdCYP73A-BamHI-Fw3 | AAATGGGTCGGGATCCGATGGATCTTCTCCTACTCGAGAAGA |
| SdCYP73A-HindIII-Rv2 | GTGCGGCCGCAAGCTTTCAAAATGATCTAGGCTTCAAGACA |
| ***Real-time analysis*** | |
| SdCPR-RT-FW2 | ATCTCGGAGGGAGGCTACAT |
| SdCPR-RT-RV2 | CTTTTTCCCGGCCATTTATT |
| SdCYP73A-UTR-FW1 | AAACATCTACGACTCCTTCCT |
| 5’-RV1 | TGTCGAATACGACGTTCCGTGTT |
| GAPDH-16-FW | ACTACACTACTCATTACTTC |
| GAPDH-16-RV | ACGTTTAATGCAATCACAAGG |
| ***Genome walking*** | |
| SdCPR-RV3 | CTGTCTGTCTTGGCTCCGCTGAAGAT |
| SdCPR5'-RV2 | TCTCTCCAAAGGGGTGGGTTTGATGA |
| GWSdCPR-FW | GCATCAAGGGCTCCAACATCACGTCTAT |
| GWSdCPR-RV | CACTGGTTAACCTTGGTTAGGTGTTAC |
| C4H-RV1 | CACGACTGAAGCGAGGACGATT |
| C4H-RV2 | GGGAGATCAGTGGTTGAGTTA |

**Table S2. GenBank ID of CPRs in Fig. 1 and Fig. 2.**

| Origin | Accession |
| --- | --- |
| *Arabidopsis thaliana* (ATR1) | AAK96879 |
| *Arabidopsis thaliana* (ATR2) | AAK17169 |
| *Capsicum annuum* | ACF17649 |
| *Catharanthus roseus* | CAA49446 |
| *Centaurium erythraea* | AAS92623 |
| *Eschscholzia californica* | AAC05022 |
| *Gossypium hirsutum* (CPR1) | ACN54323 |
| *Gossypium hirsutum* (CPR2) | ACN54324 |
| *Helianthus tuberosus* | CAB58577 |
| *Lotus japonicus* | BAG68945 |
| *Nothapodytes foetida* (CPR1) | ACF35280 |
| *Nothapodytes foetida* (CPR2) | ACF35281 |
| *Nothapodytes foetida* (CPR3) | ACF35282 |
| *Ophiorrhiza pumila* | BAC41516 |
| *Papaver somniferum* | AAC05021 |
| *Perilla frutescens* | ADC94831 |
| *Petroselinum crispum* (CPR1) | AAB97737 |
| *Petroselinum crispum* (CPR2) | AAB97738 |
| *Petunia* x *hybrida* (PR1) | AAZ39648 |
| *Petunia* x *hybrida* (PR2) | AAZ39649 |
| *Pisum sativum* (PsC450R1) | AAC09468 |
| *Populus trichocarpa* x *Populus deltoides* (CPR1) | AAK15259 |
| *Populus trichocarpa* x *Populus deltoides* (CPR2) | AAK15260 |
| *Populus trichocarpa* x *Populus deltoides* (CPR3) | AAK15261 |
| *Scoparia dulcis* | AGX85599 |
| *Solenostemon scutellarioides* | CAQ37789 |
| *Vicia sativa* | CAA81211 |
| *Vigna radiata var. radiata* | AAA34240 |
| *Withania somnifera* (CPR1) | ADI49691 |
| *Withania somnifera* (CPR2) | ADI49692 |

**Fig. S1. The reaction catalyzed by C4H in the phenylpropanoid pathway.**

PAL, phenylalanine ammonia-lyase; C4H, cinnamic acid 4-hydroxylase; 4CL, 4-coumaric acid:CoA ligase.


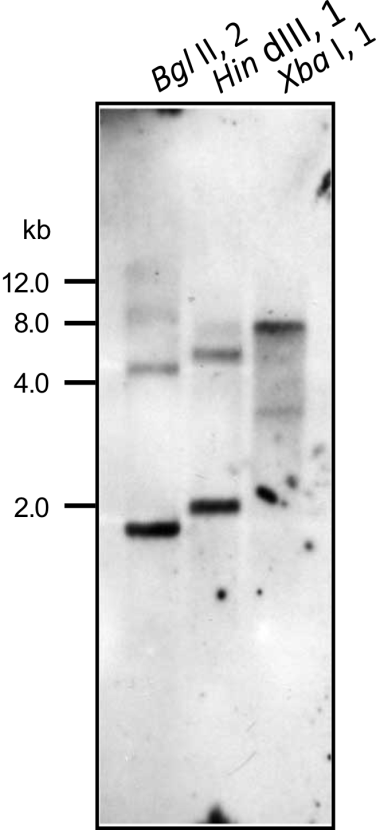


**Fig. S2. DNA blotting analysis of *SdCPR* by digesting with *Bgl*II, *Hin*dIII, and *Xba*I.**

Genomic DNA (30 μg) was digested with *Bgl*II, *Hin*dIII, and *Xba*I, separated on 0.8% agarose gels, then blotted onto a Hybond-N^+^ membrane. The membrane was hybridized with a specific probe for SdCPR. The numbers following the restriction enzyme names indicate the number of recognition sites that occur in the *SdCPR* sequence.

**Fig. S3. Immunoblotting analysis of heterologously expressed His-SdCPR (A) and His-SdC4H (B) in *E. coli*.**

The proteins were resolved by 7.5% (A) and 10.0% (B) SDS-PAGE and transferred to nitrocellulose. The immunoblots were developed with Anti-His-tag HRP-DirecT (1:10,000). M, molecular weight marker; -, total soluble fraction without IPTG; +, total soluble fraction with IPTG induction; P, purified recombinant fraction by Ni-Particles with an elution buffer containing 1 M imidazole.


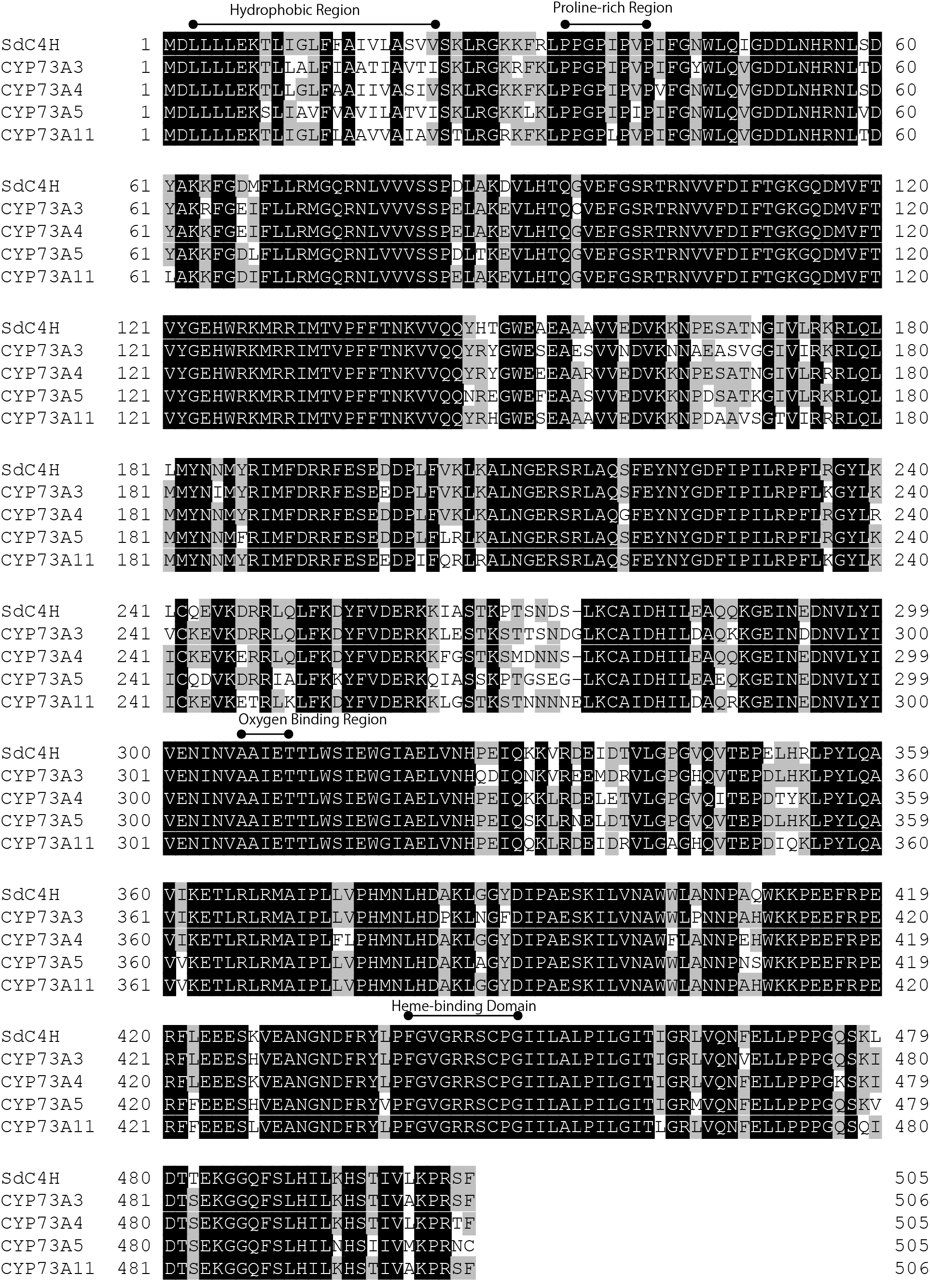


**Fig. S4. Amino acid alignment of plant CYP73A family.**

Alignment of the deduced amino acid sequences of SdC4H and plant CYP73A family members. The deduced amino acid sequences of SdC4H were aligned with those of CYP73A family members from *Medicago truncatula* (CYP73A3, ABC59087), *Catharanthus roseus* (CYP73A4, CAA83552), *Arabidopsis thaliana* (CYP73A5, AAB58356), and *Glycine max* (CYP73A11, CAA63172) using the ClustalW program. The conserved regions and binding sites are marked.


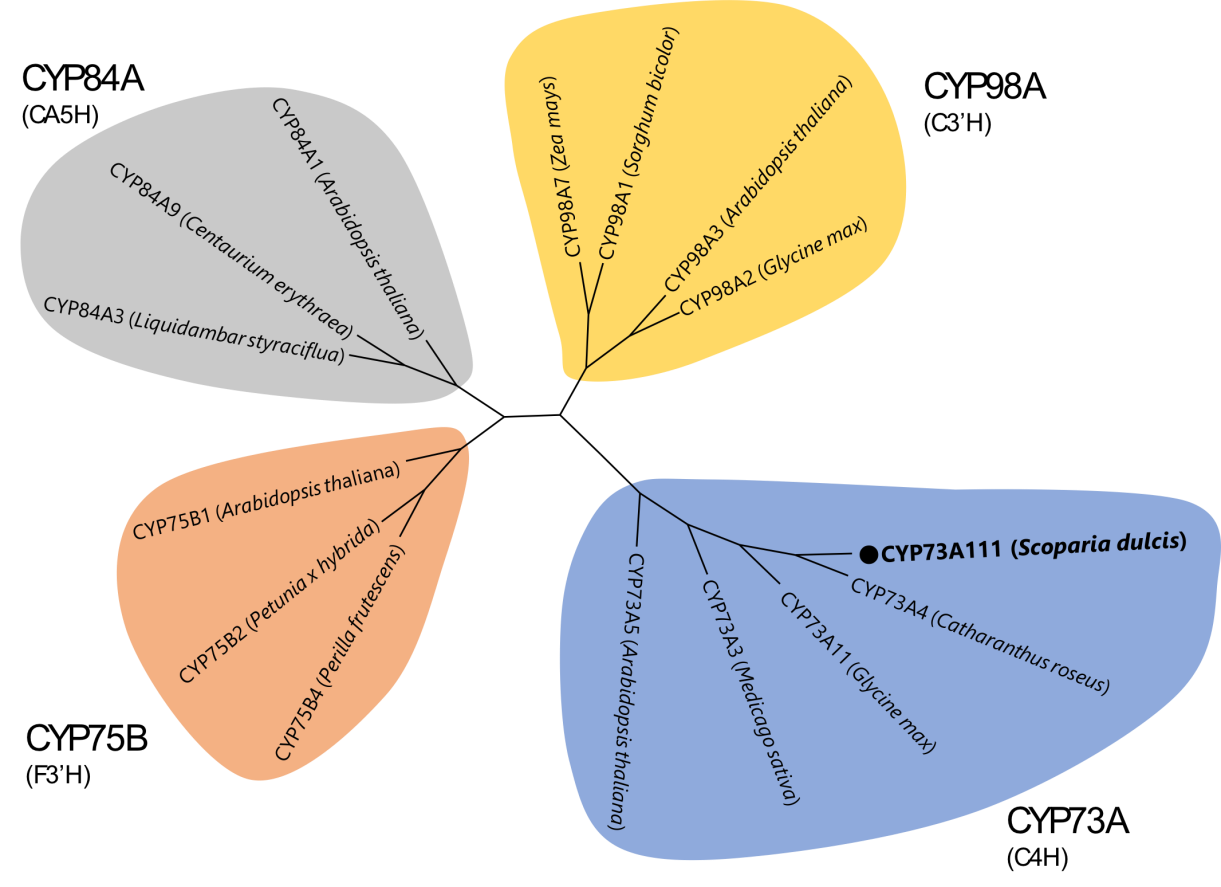


**Fig. S5. Phylogenetic tree of P450 proteins involved in the phenylpropanoid pathway.**

The maximum likelihood tree illustrates the phylogenetic relatedness of SdC4H with other P450 proteins involved in the phenylpropanoid pathway. CA5H, coniferaldehyde 5-hydroxylase; C3’H, coumarate 3-hydroxylase; F3’H, flavonoid 3’-hydroxylase; C4H, cinnamic acid 4-hydroxylase. The GenBank accession numbers for the sequences are as follows: CYP73A3 (*Medicago truncatula*, ABC59087); CYP73A4 (*Catharanthus roseus*, CAA83552); CYP73A5 (*Arabidopsis thaliana*, AAB58356); CYP73A11 (*Glycine max*, CAA63172); CYP73A111 (*Scoparia dulcis*, AGX85600); CYP75B1 (*Arabidopsis thaliana*, AAG16745); CYP75B2 (*Petunia hybrida*, AAD56282); CYP75B4 (*Perilla frutescens var. crispa*, BAB59005); CYP84A1 (*Arabidopsis thaliana*, AAC49389); CYP84A3 (*Liquidambar styraciflua*, AF139532); CYP84A9 (*Centaurium erythraea*, AAS92625); CYP98A1 (*Sorghum* *bicolor*, AAC39316); CYP98A2 (*Glycine max*, AAB94587); CYP98A3 (*Arabidopsis thaliana*, AEC09893); CYP98A7 (*Zea mays*, ACG39178). Phylogenetic analyses were performed using the Neighbor-jointing method using Genetyx ver.14 software.
